# Desertification gradients shape *Medicago sativa* rhizosphere microbiomes in inner Mongolia’s agro-pastoral ecotone

**DOI:** 10.3389/fmicb.2025.1651717

**Published:** 2025-09-22

**Authors:** Jiajia Xu, Senyuan Wang, Zhenyu Jia, Zhaoming Wang, Yuying Bao, Jie Wei

**Affiliations:** ^1^College of Life Sciences, Inner Mongolia University, Hohhot, China; ^2^Key Laboratory of Herbage and Endemic Crop Biology, Ministry of Education, Inner Mongolia University, Hohhot, China; ^3^M-Grass Ecology and Environment (Group) Co., Ltd., Hohhot, China; ^4^Inner Mongolia Engineering Technology Research Center of Germplasm Resources Conservation and Utilization, Inner Mongolia University, Hohhot, China

**Keywords:** *Medicago sativa*, rhizosphere microbiome, desertification gradients, soil texture, microbial adaptation

## Abstract

This study investigated the spatial heterogeneity of rhizosphere microbial communities in alfalfa (*Medicago sativa*) across desertification gradients in Inner Mongolia, China. Rhizosphere soils were collected from non-, lightly-, and moderately- desertified sites. Using 16S rRNA and ITS high-throughput sequencing alongside soil physicochemical analyses, we found that desertification intensity significantly altered microbial structure and function. Actinobacteriota dominated in moderately-desertified soils, whereas Proteobacteria prevailed in non-desertified areas. Ascomycota was the dominant fungal phylum, with Basidiomycota and Mortierellomycota enriched in non- and lightly- desertified sites, respectively. Soil pH and available phosphorus were the key factors shaping bacterial and fungal communities, respectively. Co-occurrence networks indicated enhanced microbial connectivity and a shift toward cooperative interactions under desertification. Functional prediction revealed conserved bacterial metabolic pathways but increased abundance of fungal stress-response enzymes (e.g., monooxygenases). These findings underscore microbial adaptive strategies to desertification and provide insights for sustainable agriculture in arid regions.

## 1 Introduction

Root-associated microbial communities are critical for plant health, nutrient cycling, and the sustainability of soil ecosystem (Berendsen et al., 2012). As core components of the rhizosphere, these microorganisms dynamically interact with plant roots, modulating soil fertility, disease resistance, and stress tolerance (Bakker et al., 2018). *Medicago sativa* (alfalfa), a globally significant leguminous forage crop, relies heavily on rhizosphere microbes for nitrogen fixation, phosphorus solubilization, and organic matter decomposition (Berendsen et al., 2012; Chamkhi et al., 2023; Liu et al., 2018). However, the composition and diversity of these microbial communities are intricately regulated by plant genotype, soil physicochemical properties, and regional environmental conditions (Bonito et al., 2019; Philippot et al., 2013). Despite extensive research on rhizosphere microbiomes, systematic investigations into the spatial heterogeneity of alfalfa-associated microbial communities in arid and semi-arid ecosystems remain scarce (Yang et al., 2021).

Inner Mongolia, a critical agro-pastoral transition zone in northern China, is also one of the regions most severely affected by soil desertification. This ecologically fragile area harbors diverse climatic zones and soil types. The Inner Mongolia region exhibits a pronounced warming-drying trend and increased climate variability. Meteorological data from 2023 to 2024 show consistently rising temperatures across the area, with 2023 being 0.9°C above the average and 2024 setting a historical record at 1.1°C higher. Precipitation patterns have also shifted significantly: while 2023 saw a 4.9% decrease (with autumn down by 16.8%), 2024 had an overall increase of 41.6%, though with uneven spatiotemporal distribution, leading to persistent drought intensification in western areas. These climatic conditions, combined with frequent severe sand and dust storms, create compound stress that accelerates regional desertification through multiple mechanisms including soil wind erosion, nutrient loss, and vegetation destruction (National Climate Center, 2024, 2025).

Alfalfa cultivation in this region is vital not only for livestock fodder production and soil conservation (Feng et al., 2022), but also as a promising strategy for combating desertification. However, local soils face escalating threats from degradation, salinization, and nutrient depletion, necessitating a deeper understanding of soil-microbe-plant interactions to inform sustainable management practices (Jing et al., 2022; Li Y. et al., 2023; Semchenko et al., 2022). Previous studies have demonstrated that microbial communities are highly sensitive to environmental shifts caused by desertification, such as changes in soil pH, organic carbon, and nutrient availability (Fierer and Jackson, 2006; Rousk et al., 2010; Tian et al., 2021). For instance, alkaline soils in arid regions favor Actinobacteria, while acidic soils enrich Acidobacteria (Lauber et al., 2009). Nevertheless, multi-site comparative studies integrating high-throughput sequencing to unravel how desertification gradients reshape the spatial heterogeneity of alfalfa rhizosphere bacteria and fungi in this region are still lacking.

This study focuses on rhizosphere soils of *M. sativa* from four distinct regions in Inner Mongolia: Tongliao (Zhuruhe River and Xinli Farm), Hohhot (Horinger County), and Bayannur (Urad-middle-qi). These regions exhibit gradients in climate, soil texture, and land-use history, providing an ideal framework to explore how soil factors drive microbial community assembly (Drenovsky et al., 2009; Kostin et al., 2021). By combining molecular techniques (16S rRNA and ITS sequencing) with comprehensive soil physicochemical analyses (organic matter, nitrogen, phosphorus, potassium, and pH), we address three key questions: How do bacterial and fungal communities in alfalfa rhizospheres differ across agroecological zones in Inner Mongolia? Which soil parameters are most strongly associated with variations in microbial diversity and composition? Do regional differences in soil properties override plant-specific effects as the dominant drivers of rhizosphere microbiome assembly?

Our findings aim to optimize alfalfa cultivation strategies, enhance soil health, and improve microbial-driven resilience in vulnerable ecosystems. This work advances theoretical insights into rhizosphere ecology in arid regions and provides actionable guidelines for sustainable agriculture and ecological restoration.

## 2 Materials and methods

### 2.1 Sample design

The study area is in a typical agro-pastoral ecotone of Inner Mongolia Autonomous Region, covering three representative regions: Tongliao City (Zhurihe River and Jarud-qi), Hohhot City (Horinger County), and Bayannur City (Urad-middle-qi). This region belongs to a temperate continental monsoon climate zone, with an annual precipitation of 350–450 mm and an average annual temperature of 5°C–7°C, exhibiting distinct arid to semi-arid characteristics (Lv et al., 2021; Supplementary Table 2). A systematic sampling strategy was adopted to collect 18 rhizosphere soil samples during the vigorous growth period of *Medicago sativa* (September 2023). Sampling sites included six representative plots: Zhurihe River (ZRHA, ZRHC), Jarud-qi (ZLT), Horinger County (HLA, HLB), and Urad-middle-qi (WLT). This period corresponds to the annual peak activity of the alfalfa rhizosphere microbial community.

Based on soil texture analysis (Supplementary Figure 1), the soils across the sampling sites were classified into three categories: semi-hydromorphic soil, pedocals, luvisols (FAO, 2015). Notably, these sites represent three desertification gradients (non-desertified, lightly-desertified and moderately-desertified; Supplementary Figure 1) and Microbial community similarity across a desertification gradient (Supplementary Figure 2), providing an ideal framework to investigate rhizosphere microecological characteristics of alfalfa under varying desertification intensities. The selection of sampling sites fully accounted for regional climatic gradients (annual precipitation decreasing from 450 mm in the east to 350 mm in the west) and representative land-use patterns, ensuring the ecological relevance of the findings (Lv et al., 2021).

### 2.2 Soil sample collection

To ensure representative sampling of rhizosphere soils, three 10m × 10m quadrats were established per plot. Healthy alfalfa plants exhibiting vigorous growth were randomly selected, and their entire root systems (depth: 20–30 cm, encompassing the primary root zone) were carefully excavated using a sterilized stainless-steel shovel at 10–15 cm from the plant base. Non-rhizosphere soil was gently removed by manual shaking, retaining only the root-adhered soil. A minimum of nine plants per quadrat were pooled to form a composite rhizosphere soil sample. Each plot yielded five homogenized subsamples, which were combined into three composite samples per plot, resulting in a total of 18 composite samples across six study plots. This replication scheme ensured statistical robustness for subsequent analyses. Samples were immediately flash-frozen on dry ice, transferred to liquid nitrogen storage within 2 h, and maintained at −80°C until laboratory processing for physicochemical characterization and high-throughput sequencing.

### 2.3 Soil physical and chemical determination

Soil organic matter (SOM) and soil organic carbon (SOC) were determined by dichromate oxidation (Reigosa et al., 2015). Alkali-hydrolyzable nitrogen (AN) were measured via spectrophotometry following extraction with a potassium chloride solution (Zhang et al., 2021a). Soil available phosphorus (SAP) in soils was extracted using Hydrogen carbonate extraction - molybdenum-antimony anti-spectrophotometric method (De Silva et al., 2015). Total phosphorus (tP) is extracted using a digestion method involving HCl_4_O-H_2_SO_4_ for colorimetric analysis (Zhao et al., 2018). Exchangeable potassium (AK) and Total potassium (tK) was analyzed via flame photometry after ammonium acetate extraction (Ma et al., 2020). Soil pH was measured using a calibrated pH meter (1:2.5 soil-to-water suspension) (Guo et al., 2020).

### 2.4 DNA extraction and PCR amplification

Microbial genomic DNA was extracted from 0.5 g of each soil sample using the FastDNA SPIN Kit for Soil (MP Biomedicals, Solon, OH, USA) according to the manufacturer’s protocol. The V3–V4 hypervariable regions of the bacterial 16S rRNA gene were amplified with the primers 338F (5′-ACTCCTACGGGAGGCAGCAG-3′) and 806R (5′-GGACTACHVGGGTWTCTAAT-3′). The ITS1 region of the fungal rRNA gene locus was amplified with the primers ITS1F (5′-CTTGGTCATTTAGAGGAAGTAA-3′) and ITS2R (5′-GCTGCGTTCTTCATCGATGC-3′) (Parada et al., 2015). The DNA concentration and purity were determined utilizing a NanoDrop ND-1000 spectrophotometer (NanoDrop Technologies, Wilmington, DE, USA), and extracted DNA quality was tested with 1% agarose gel electrophoresis (5 V/cm, 20 min) (Nan et al., 2020). The amplified products were sequenced on an Illumina MiSeq platform using a 2 × 300 bp (600 cycles) reagent kit at Majorbio Co., Ltd., (Shanghai, China). The Illumina MiSeq sequencing data was sent to the Sequence Read Archive (SRA) database of the National Center for Biotechnology Information (NCBI), with accession number PRJNA1303413 (SRR34926142-SRR34926147) for prokaryote and PRJNA1303431 (SRR34925753-SRR34925758) for fungi.

### 2.5 Statistical analyses

Raw sequencing data were processed using the DADA2 pipeline within QIIME 2 (version 2024.10). Briefly, primer sequences were trimmed using the cutadapt plugin. Reads were then quality-filtered, denoised, merged, and chimeras were removed using the DADA2 algorithm, resulting in amplicon sequence variants (ASVs). Taxonomy was assigned to ASVs against the SILVA reference database (version 138.2).

Statistical analyses were conducted using R software (version 3.6) and IBM SPSS Statistics 27.0. Kruskal-Wallis in SPSS was employed to evaluate significant differences (*p* < 0.05). Microbial community visualization was achieved through Circos diagrams generated with the R package Circlize, illustrating genus-level distributions of dominant bacterial and fungal taxa. Taxon-specific biomarkers were identified using the LEfSe method with Galaxy platform (LDA score > 4.0, Benjamini-Hochberg FDR, *q* < 0.05)^1^. β-diversity patterns were assessed via principal coordinate analysis. Principal coordinate analysis (PCoA) of weighted Bray-Curtis distances was conducted with PERMANOVA and BETADISPER analyses. Co-occurrence networks were generated via the iNAP web server^2^ employing SPRCC analysis (|ρ| > 0.6, *p* < 0.05) for network construction and topological feature analysis, with final visualizations optimized in Gephi (v0.10.1). Relationships between microbial communities and edaphic factors were explored through Mantel tests and Pearson correlations (ggplot2 and linkET packages). After processing physicochemical data with VIF (VIF < 5: Collinearity is not severe; 5 ≤ VIF < 10: Moderate multicollinearity exists; VIF ≥ 10: Indicates severe multicollinearity) and VPA (Variance Partitioning Analysis, VPA), the relationship between microbial communities and soil environmental factors was revealed using constrained redundancy analysis (RDA). Bacterial functional profiles and fungal functional profiles were predicted via PICRUSt2 based on normalized ASV tables and NSTI scores. The Majorbio Cloud Platform facilitated phylum-level taxonomic profiling and alpha diversity calculations (Chao, Shannon, Shannon-even).

## 3 Result

### 3.1 Response of rhizosphere soil microbial communities in alfalfa to desertification gradients

**FIGURE 1:**
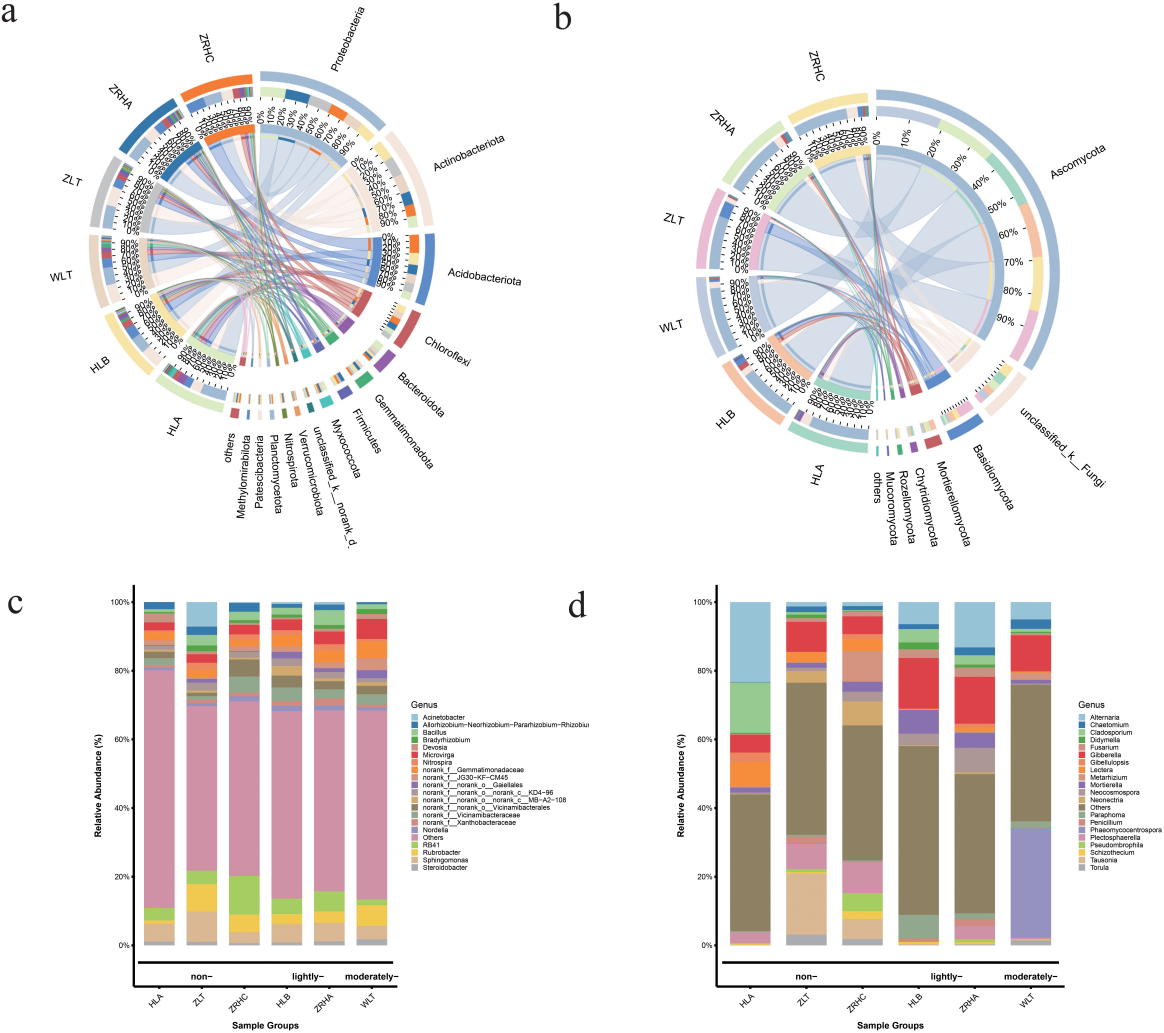
Root-associated microbial composition of alfalfa across six regions. **(a)** Bacterial phylum-level composition; **(b)** fungal phylum-level composition; **(c)** bacterial genus-level composition; **(d)** fungal genus-level composition.

As soil desertification intensifies, the structure of rhizosphere soil microbial communities in alfalfa undergoes significant changes. Bacterial community analysis reveals (Figure 1a) that the relative abundance of Actinobacteriota shows a positive correlation with the degree of desertification, increasing significantly from 16.39% in non-desertified site HLA to 27.73% in moderately-desertified site WLT. In contrast, Proteobacteria dominates in non-desertified site HLA (37.64%) but decreases markedly to 24.22% in lightly desertified site HLB. Notably, Acidobacteriota exhibits its highest relative abundance (27.00%) in non-desertified site ZRHC. The fungal community is predominantly composed of Ascomycota, maintaining high relative abundances in both non-desertified site HLA (78.68%) and moderately-desertified site WLT (85.49%). Basidiomycota is significantly more abundant in non-desertified site ZLT (20.55%) compared to other sites (approximately 2–6 times higher), while Mortierellomycota displays a distinct gradient pattern, peaking in lightly-desertified site HLB (7.25%) and dropping to its lowest level in moderately-desertified site WLT (1.17%) (Figure 1b).

At the genus level, microbial communities exhibit even more pronounced responses to desertification (Figures 1c, d). In bacterial communities, *unclassified_f__Micrococcaceae* shows significantly higher abundance in lightly-desertified site HLB (8.37%) than in non-desertified site HLA (1.49%). Non-desertified site ZRHC is characterized by the dominance of *RB41* (7.89%), while ZLT is enriched with *Sphingomonas* (6.90%) and *Acinetobacter* (4.70%). Among fungal communities, *Gibberella* is significantly more abundant in HLB (14.73%) than in HLA (5.20%). The distribution of *Alternaria* varies markedly, peaking in HLA (23.26%) and dropping to only 1.16% in ZRHC. Notably, *Phaeomycocentrospora* dominates in moderately-desertified WLT (31.84%), while *Tausonia* exhibits a unique distribution pattern, with high abundance detected only in ZLT (17.84%). Additionally, *Mortierella* is significantly more abundant in HLB (6.87%) than in HLA (1.47%). These distinctive distribution patterns of characteristic microbial genera clearly reflect the adaptive evolution of rhizosphere micro-ecosystems to varying degrees of desertification.

**FIGURE 2:**
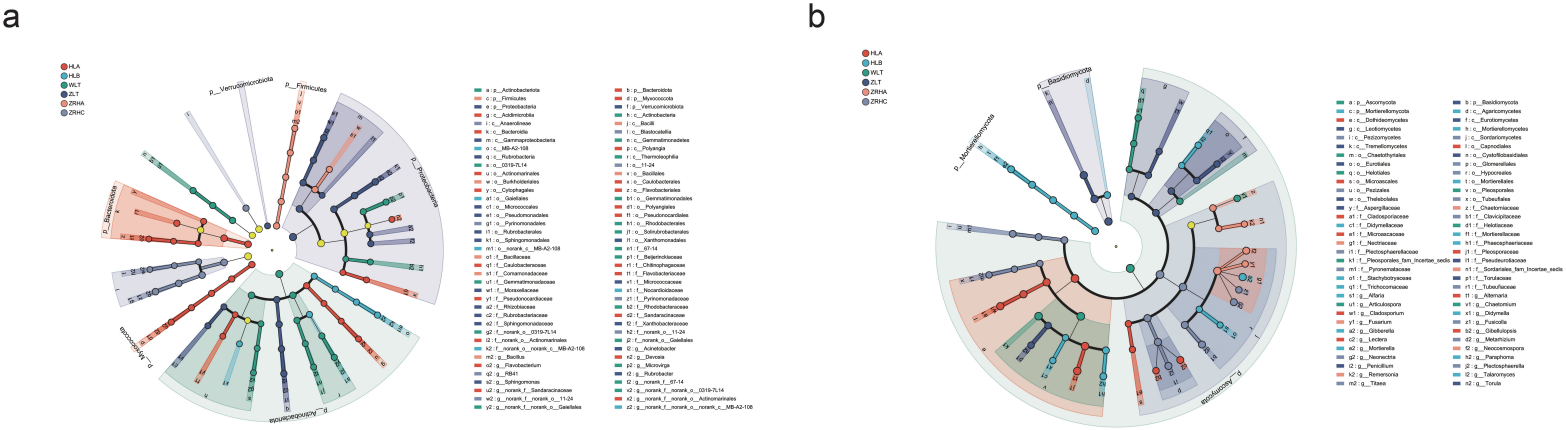
LEfSe analysis of rhizosphere microbial communities in alfalfa across different regions (p, phylum; c, class; o, order; f, family; g, genus). **(a)** Cladogram shows the taxonomic distribution of biomarker taxa across hierarchical levels in bacterial sample groups; **(b)** cladogram shows the taxonomic distribution of biomarker taxa across hierarchical levels in fungal sample groups. Multiple testing correction was performed using the Benjamini-Hochberg FDR, *q* < 0.05.

LEfSe analysis (LDA scores > 4, *q* < 0.05) identified 117 significantly differentiated microbial taxa (51 bacterial and 66 fungal) that exhibited clear gradient distribution patterns along the desertification gradient (Figure 2, Supplementary Figure 4). Bacterial community analysis demonstrated specific enrichment of Bacteroidota and Myxococcota in non-desertified HLA plots, while Actinobacteriota emerged as the characteristic phylum in moderately-desertified WLT plots. Proteobacteria and Firmicutes were distinctly associated with non-desertified ZLT and lightly-desertified ZRHA plots, respectively. At the genus level, *norank_f__Sandaracinaceae* (HLA), *norank_f__norank_o__norank_c__MB-A2-108* (HLB), and *norank_f__norank_o__Gaiellales* (WLT) were identified as key biomarker taxa. Fungal community analysis revealed Ascomycota as the dominant phylum in moderately-desertified WLT plots, with Basidiomycota and Mortierellomycota showing significant enrichment in non-desertified ZLT and lightly-desertified HLB plots, respectively. Genus-level analysis showed specific enrichment of *Alternaria* in HLA plots, ubiquitous distribution of *Gibberella* across all sites, and significantly higher abundance of *Chaetomium* in ZRHA plots. These gradient distribution patterns not only demonstrate the strong bioindicator potential of specific microbial taxa but also reflect the ecological strategies employed by rhizosphere microbial communities to adapt to varying desertification stress through functional group reorganization.

### 3.2 Characteristics of rhizosphere microbial diversity in alfalfa soils across desertification gradients

**FIGURE 3:**
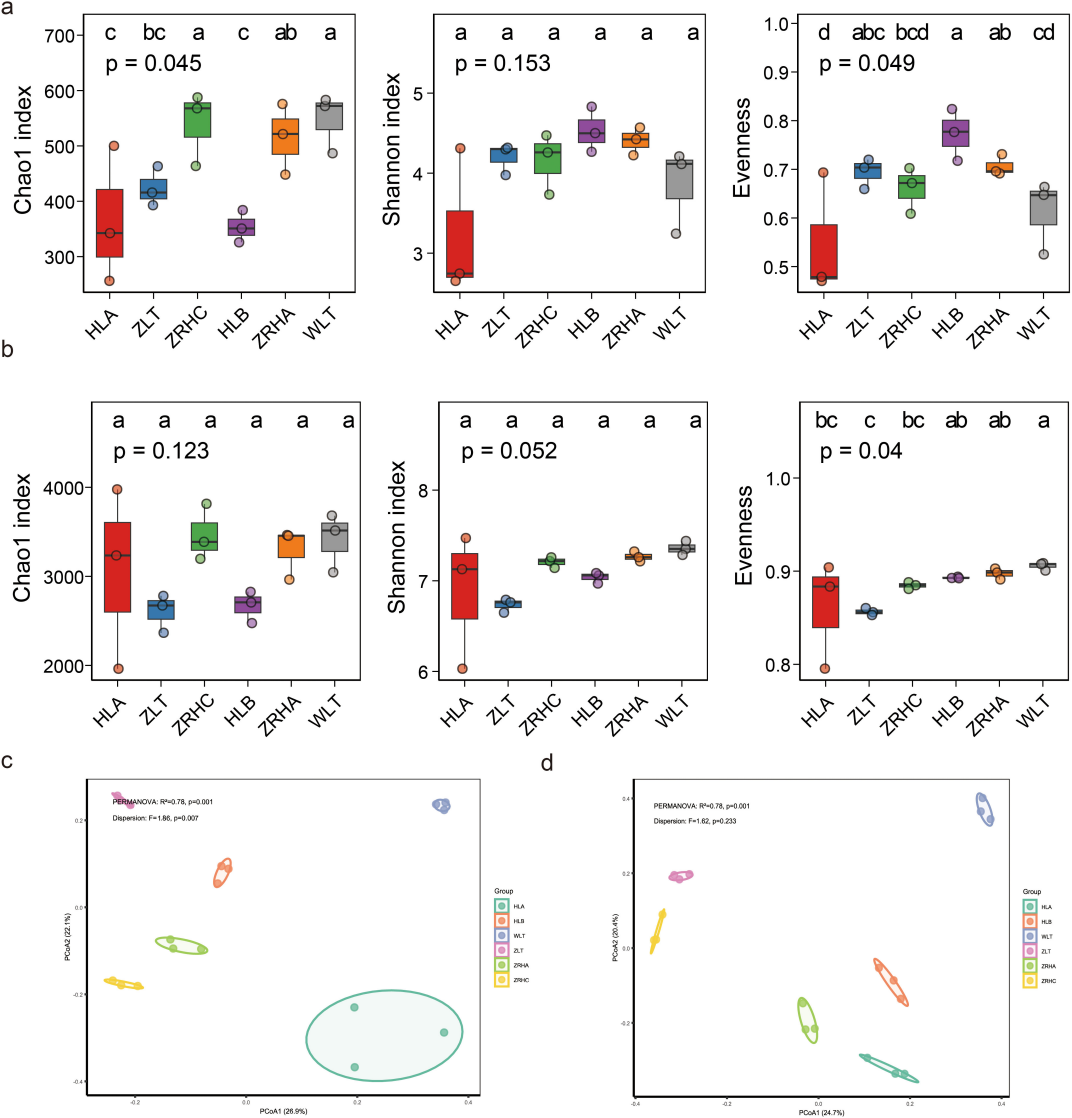
Analysis of rhizosphere microbial communities in 6 regions. **(a)** α diversity analysis of bacterial communities, showing: Chao index, Shannon index, and evenness index. **(b)** α diversity analysis of fungal communities, showing: Chao1 index, Shannon index, and evenness index; **(c)** PCoA Bray-Curtis distance-based analysis of bacterial communities; **(d)** PCoA Bray-Curtis distance-based analysis of fungal communities. The statistical significance was tested using the Kruskal-Wallis method, *p* < 0.05, values with different lowercase letters are significantly different among plots. The result indicates significant differences in microbial community composition between groups. (PERMANOVA, *p* < 0.05, BETADISPER, *p* > 0.05).

The ASV rarefaction curves indicated that the sequencing depth was sufficient to support subsequent analyses (Supplementary Figure 3). Alpha diversity analysis revealed no significant differences in bacterial community diversity (Chao1, Shannon) across desertification gradients, indicating their strong environmental adaptability. In contrast, fungal communities exhibited significant variations in diversity indices (Chao1, Evenness), with notably lower diversity observed in the non-desertified HLA plots (*p* < 0.05) (Figures 3a, b).

Principal Coordinates Analysis (PCoA) based on weighted Bray-Curtis distances showed that PC1 and PC2 explained 26.9% and 22.1% of bacterial community variation, and 24.7% and 20.4% of fungal community variation, respectively (Figures 3c, d). Microbial community structures showed significant clustering patterns among sampling sites, with particularly similar structures observed among lightly-desertified (HLB, ZRHA) and non-desertified (ZRHC) plots, likely reflecting their environmental consistency in soil type and vegetation composition. Notably, the moderately-desertified WLT plots exhibited distinct community structures that significantly diverged from other sites, further highlighting the predominant role of desertification intensity in driving rhizosphere microbial community assembly in alfalfa. PCoA of the bacterial community along a Normalized Difference Vegetation Index (NDVI)-based desertification gradient revealed that the microbial community composition in moderately-desertified areas was significantly distinct from other regions (Supplementary Figure 2). Specifically, the microbial composition exhibited certain similarities between non-desertified and lightly-desertified areas. The first two principal coordinates (PC1 and PC2) collectively explained 45.03% of the total variation in the bacterial community and 45.01% in the fungal community. Specifically, PC1 accounted for 22.94% of the variance in bacteria and 24.66% in fungi, clearly separating the moderately-desertified samples from the others in both communities.

### 3.3 Characteristics of rhizosphere microbial co-occurrence networks in alfalfa across desertification gradients

**FIGURE 4:**
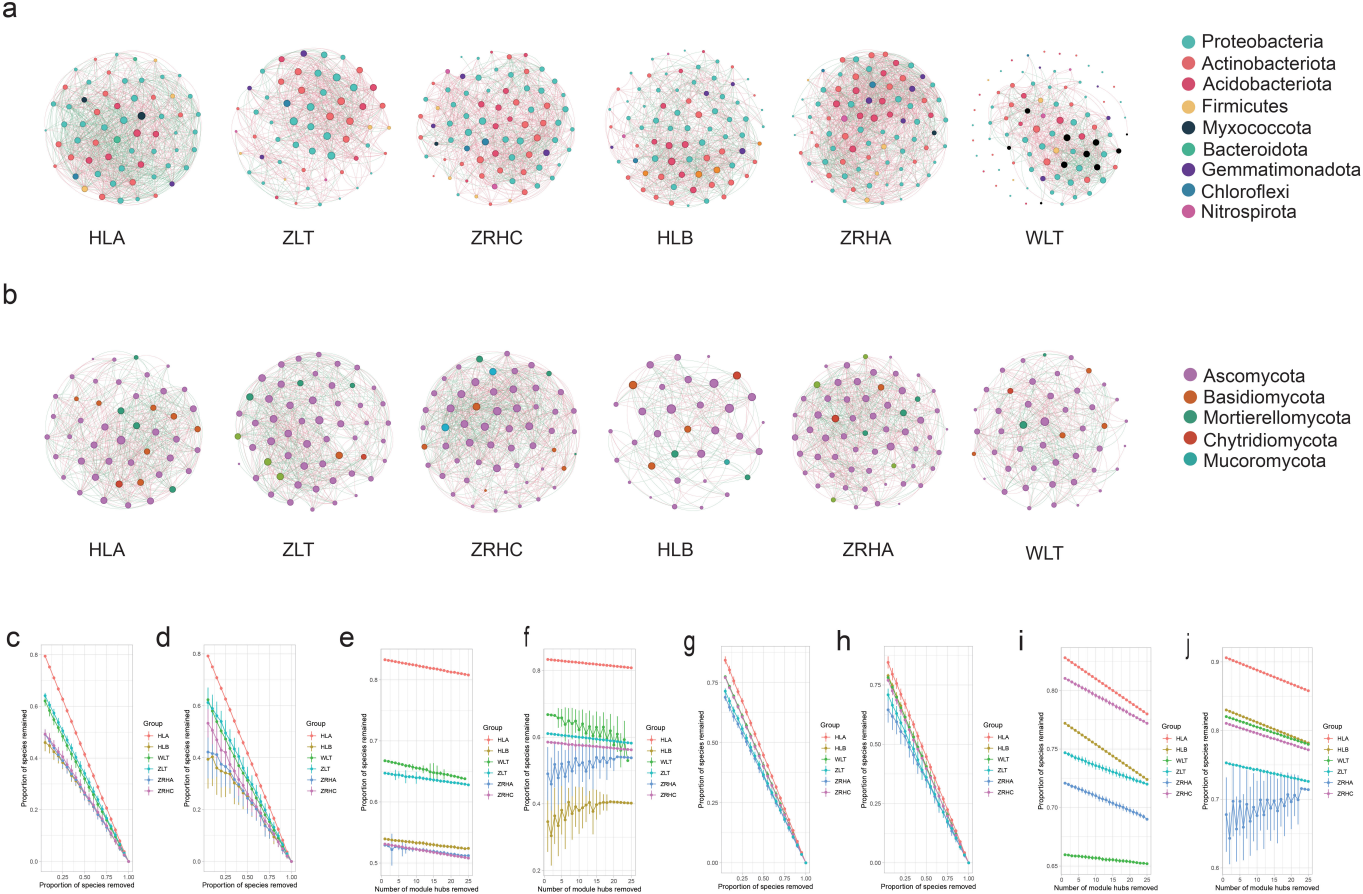
Microbial co-occurrence networks for six sample sites (HLA, ZLT, ZRHC, HLB, ZRHA, WLT). Panel **(a)** shows bacterial networks with colored nodes representing different phyla. Panel **(b)** shows fungal networks with nodes colored by phylum. Panels **(c–f)** present bacterial network stability and robustness analysis under different node-removal scenarios: random removal, targeted removal of keystone taxa, and removal of module hubs. Panels **(g–j)** show fungal network stability under the same removal strategies. Network significance thresholds: Edges: SparCC |ρ|> 0.6 & *p* < 0.05, Keystone nodes: Degree > 10 & Betweenness centrality > 100.

To construct topological networks, we selected the 100 most abundant ASV from both the bacterial and the fungal datasets. Microbial co-occurrence network analysis revealed significant differences in interaction patterns among rhizosphere microbial communities of alfalfa across desertification gradients (Figure 4, Supplementary Tables 2, 3). In bacterial networks, non-desertified HLA and lightly-desertified ZRHA plots exhibited the highest connectivity densities (32.355 and 29.881, respectively), while moderately-desertified WLT showed the lowest density (13.64). The proportion of positive interactions was highest under moderate desertification (ZRHA, 62.17%) but remained elevated in severe desertification (WLT, 61.63%) compared to light desertification (HLA, 52.74%), suggesting a general shift toward more cooperative microbial interactions under environmental stress.

Fungal networks displayed distinct response patterns, with the highest connectivity density in non-desertified ZRHC (28.60) and the lowest in lightly-desertified HLB (11.83). The co-occurrence network derived from the HLB plot exhibited the sparsest architecture among all sampled sites (|ρ| > 0.6, *p* < 0.05). The ratio of positive to negative interactions remained relatively balanced across sites, except for slightly higher positive interactions in ZRHA (59.57%). Network modularity analysis showed bacterial communities reached peak modularity in HLB (0.272) and ZRHC (0.294) plots, whereas fungal networks maintained relatively consistent modularity across sites (0.163–0.251). Furthermore, network robustness analysis showed that the stability of all networks heavily relied on the presence of keystone taxa, providing new insights into the stability mechanisms of microbial communities in desertified ecosystems.

### 3.4 Response of alfalfa rhizosphere soil microbial communities to environmental factors across desertification gradients

**FIGURE 5:**
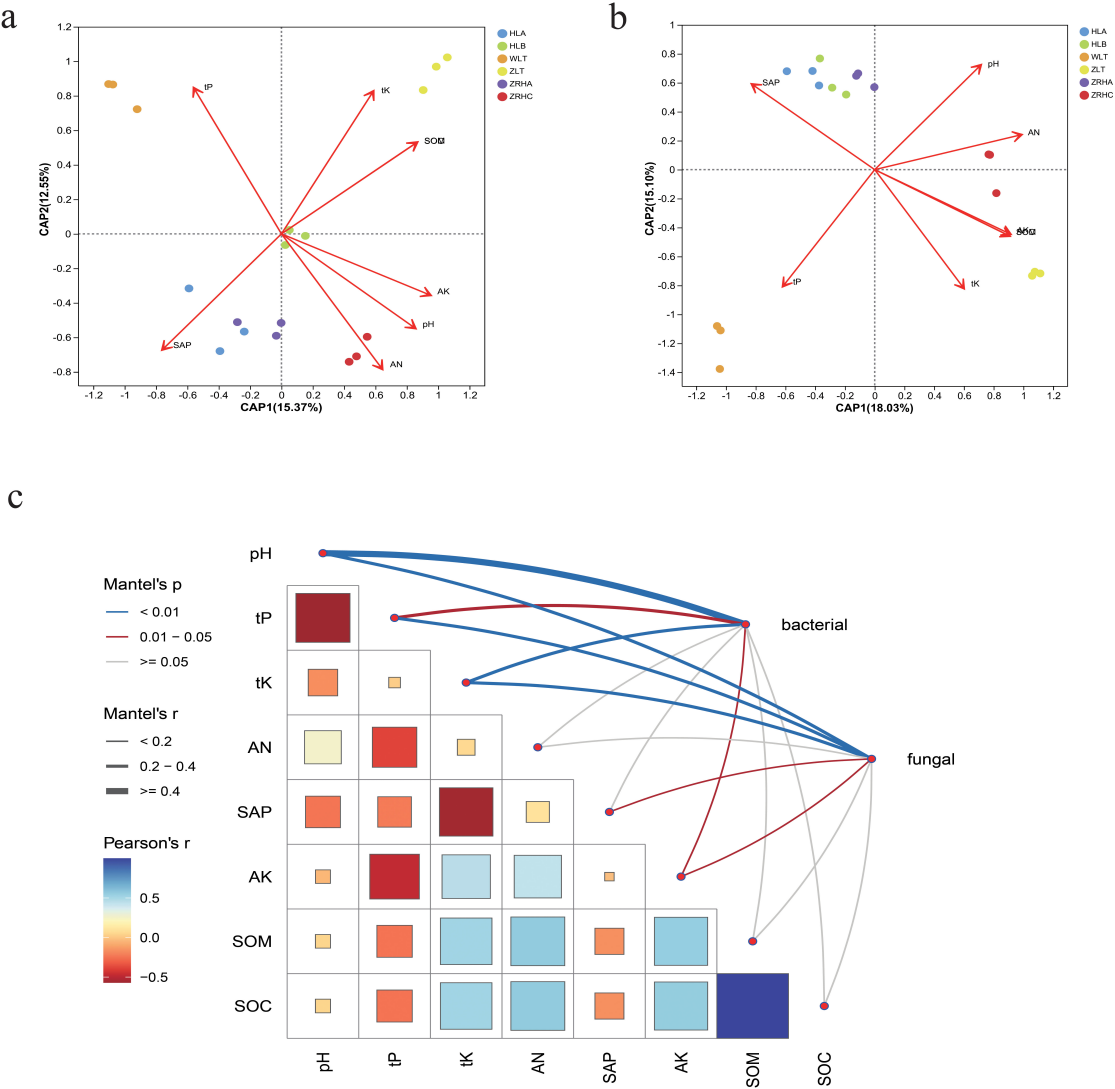
Multi-scale driving mechanism of soil physicochemical properties on rhizosphere microbial communities. **(a)** db-RDA analysis of the effects of soil physiochemistry on bacterial communities in different regions; **(b)** db-RDA analysis of the effects of soil physiochemistry on fungal communities in different regions; **(c)** mantel-test analysis of the effects of soil physiochemistry on microbial communities.

Soil physicochemical properties exhibited significant variations among different desertification gradients (*p* < 0.05). Prior to multivariate analysis, variance inflation factor (VIF) analysis was performed to assess multicollinearity among soil chemical parameters. All retained variables had VIF values below 5, indicating acceptable levels of collinearity for reliable interpretation of the regression models (Supplementary Table 5). The moderately-desertified WLT plots were distinctly acidic (pH 6.69 ± 0.16), significantly lower than other alkaline sites (Supplementary Table 4). Variance partitioning analysis (VPA) revealed that the tested environmental factors together explained 33% of the total variation in the bacterial community (Supplementary Figure 5). Among them, soil pH had the largest independent contribution, explaining 10% of the variation alone. SAP independently explained 9% of the variation. For the fungal community, the total explanatory power of environmental factors was lower, at 28%, but the key drivers were different from those for bacteria. SAP was the primary driver for the fungal community, with an independent contribution of 9%. Notably, the lightly-desertified ZRHA plots contained the highest available phosphorus content, while ZRHA and non-desertified ZRHC plots were significantly richer in available potassium (Figures 5a, b). Although the non-desertified ZLT plots showed the highest total potassium and nitrogen contents, they displayed the lowest available phosphorus levels. Importantly, ZLT, ZRHC and ZRHA plots all maintained significantly higher soil organic carbon (SOC: 12.4–15.2 g ⋅ kg^–1^) and C/N ratios (14.3–16.8) compared to other sites.

Correlation analysis between microbial communities and environmental factors demonstrated significant impacts of desertification gradients on distribution patterns (Figure 5c). Microbial communities in moderately-desertified areas showed significant positive correlations with total phosphorus, whereas non-desertified areas were more influenced by total potassium, organic matter and organic carbon. Bacterial communities were primarily affected by available phosphorus, available nitrogen and pH, while fungal communities exhibited more complex responses with site-specific associations with nutrients and pH. Multivariate analysis confirmed that desertification gradients significantly influenced microbial community structure and function through key environmental factors. Mantel tests indicated soil total phosphorus (tP) negatively correlated with bacterial communities (*p* < 0.05) but positively influenced fungal communities (*p* < 0.05). As a core regulatory factor, pH demonstrated significant positive effects on both bacterial and fungal communities (*p* < 0.01), highlighting its dual regulatory role.

### 3.5 Functional differentiation of rhizosphere microbial communities in alfalfa across desertification gradients

**FIGURE 6:**
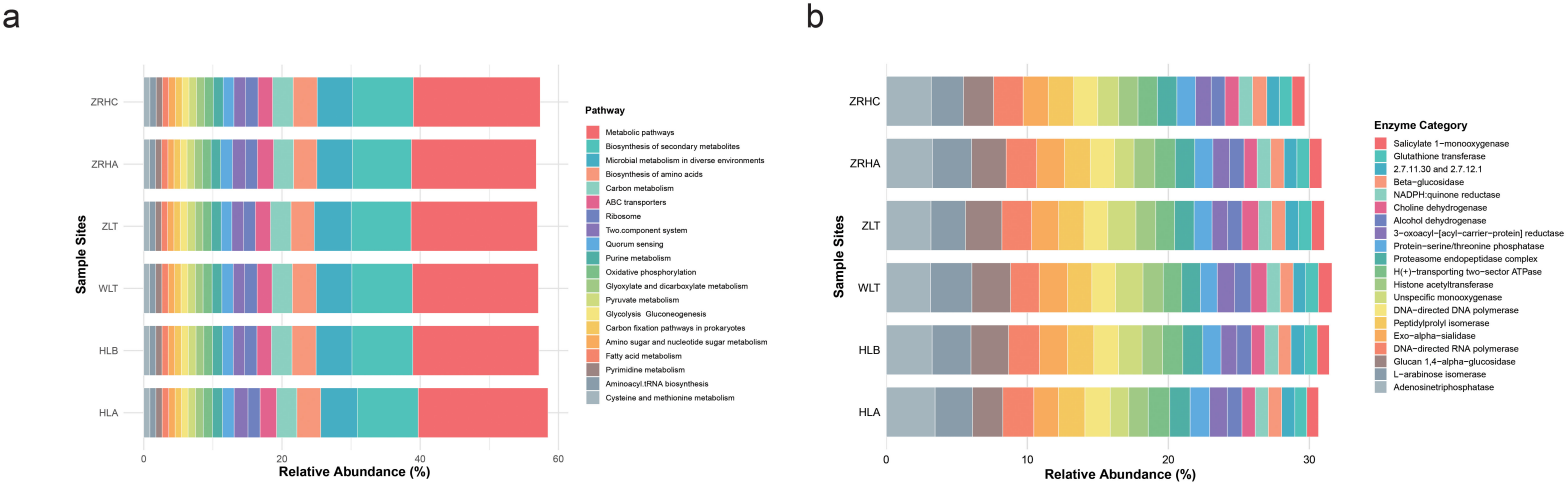
Functional prediction of bacterial and fungal communities in alfalfa rhizosphere soils. **(a)** Bacterial functional profiles predicted by PICRUSt2; **(b)** Fugal functional profiles predicted by PICRUSt2.

Functional prediction analyses revealed that desertification gradients significantly shaped microbial metabolic characteristics (Figure 6). Analysis of the relative abundance of bacterial metabolic pathways revealed a high degree of functional conservation across the six sampled sites. The overarching “Metabolic pathways” category was the most abundant, with values ranging from 18.07% (light-desertified ZRHA) to 18.77% (non-desertifed HLA). This was followed by “Biosynthesis of secondary metabolites” (8.52%–8.82%) and “Microbial metabolism in diverse environments” (5.15%–5.35%). While the overall functional profiles were similar, some variation was observed in specific pathways. The relative abundance of “ABC transporters” was highest in the HLA site (2.39%) and lowest in the HLB site (2.08%). In contrast, the relative abundances of core metabolic pathways such as “Biosynthesis of amino acids,” “Carbon metabolism,” “Glycolysis/Gluconeogenesis,” “Oxidative phosphorylation,” “Purine metabolism,” and “Pyruvate metabolism” showed minimal variation across all sites.

For the fungal communites, dominated by Adenosinetriphosphatase (3.1%–3.5%), DNA-directed RNA polymerase, and DNA-directed DNA polymerase, indicating stable investment in energy metabolism and genetic information processing. The most significant variation was observed in enzymes associated with stress response and detoxification. The relative abundance of Unspecific monooxygenase was markedly elevated in the moderately-desertified WLT (1.94%) and non-desertified ZLT (1.99%) sites compared to others (e.g., 1.32% in HLA). A congruent enrichment pattern was identified for Choline dehydrogenase (ZLT: 1.18%, WLT: 1.13%) and Salicylate 1-monooxygenase (WLT: 0.97%). Additionally, several carbohydrases, including L-arabinose isomerase and Glucan 1,4-alpha-glucosidase, were most abundant in the WLT site (2.92% and 2.74%, respectively). The pronounced enrichment of specific monooxygenases, dehydrogenases, and carbohydrasesindicates a site-specific enhancement of metabolic pathways for detoxification and the breakdown of complex substrates, likely reflecting an adaptive response to local environmental conditions.

## 4 Discussion

### 4.1 Response of microbial diversity and community structure to desertification stress

This study revealed the response characteristics of rhizosphere microbial communities in alfalfa to desertification gradients through multi-angle analysis. Bacterial communities demonstrated strong environmental adaptability, as evidenced by stable α-diversity indices (Chao, Shannon) across different desertification gradients, suggesting their potential to maintain community stability through robust environmental adaptation capabilities (Huang et al., 2024; Kuang et al., 2022; Mougi, 2024; Wang et al., 2022). In contrast, fungal communities exhibited higher sensitivity to desertification stress, with not only significant variations in alpha diversity indices (Chao1, Evenness) but also significantly reduced diversity observed in non-desertified HLA plots (*p* < 0.05); PCoA analysis further demonstrated more pronounced structural responses of fungal communities to drought stress, with PC1 and PC2 explaining 24.7% and 20.4% of the variation, respectively (de Vries et al., 2018).

The bacterial community exhibited a marked increase in Actinobacteriota abundance with intensifying desertification (non-desertified site HLA: 16.39% → moderately-desertified site WLT: 27.73%). This competitive advantage stems from their oligotrophic adaptability and drought-resistant mechanisms, including spore formation and secretion of hydrolytic enzymes such as chitinases (Li et al., 2025; Pantigoso et al., 2025). In contrast, Proteobacteria showed a sharp decline in abundance at the moderately-desertified site HLB (24.22% vs. HLA 37.64%), reflecting this phylum’s heightened sensitivity to variations in carbon source types and availability (Kim et al., 2021). At the genus level, microbial distribution patterns further demonstrated the influence of desertification gradients. For instance, *unclassified_f__Micrococcaceae* reached peak abundance at the moderately-desertified HLB site, likely associated with its drought-tolerant characteristics (Zhang et al., 2024a).

The fungal communities displayed distinct niche differentiation patterns. Ascomycota maintained dominance across all gradients (HLA: 78.68%; WLT: 85.49%) through their broad substrate utilization capacity (Yang et al., 2023), while Mortierellomycota showed significant enrichment at the moderately-desertified HLB site (7.25%), potentially alleviating nutrient limitation stress during initial desertification stages via organic phosphorus mineralization (Wachowska and Rychcik, 2022; Zhang et al., 2021b). Genus-level differentiation was particularly pronounced: fungal genera including *Gibberella* and *Alternaria* exhibited substantial variation among sites, likely reflecting differential responses of their ecological functions (e.g., phytopathogenicity, saprophytism) to environmental changes (Luo et al., 2025; Martiny et al., 2023). The absolute dominance of Phaeomycocentrospora at WLT (31.84%) underscores its ecological role as a pioneer genus in desertified conditions, while *Mortierella*’s elevated abundance at HLB (6.87%) corroborates phylum-level observations, collectively establishing a functional synergy network of “nutrient activation-stress response” (Ping et al., 2024; Qin et al., 2024).

The 117 gradient-specific biomarkers (51 bacterial and 66 fungal) identified by LEfSe analysis provide a blueprint for functional differentiation: The enrichment of Bacteroidota and Myxococcota in non-desertified HLA drives complex organic matter degradation, supporting decomposition functions in high-resource environments (Padfield et al., 2024; Wu et al., 2021). Meanwhile, the co-enrichment of Actinobacteriota and Gaiellales in moderately desertified WLT, along with the proliferation of *unclassified_f__Micrococcaceae* in HLB (8.37%), collectively enhances stress-resistant metabolic pathways (Metze et al., 2023; Zhang et al., 2019). In moderately-desertified areas, the synergistic presence of Firmicutes (ZRHA) and Mortierellomycota (HLB) employs a coordinated strategy to cope with initial stress conditions (Liu et al., 2025). We hypothesize that the distribution of these microbial taxa reflects a functional restructuring of the community in response to resource limitations driven by desertification, transitioning from a “decomposition-dominant” regime to a “stress resistance-dominant” configuration (Romdhane et al., 2022).

### 4.2 Soil heterogeneity drives microbial interaction network evolution through resource allocation

This study reveals the structural evolution of rhizosphere microbial interaction networks in alfalfa under desertification gradients and their environmental driving mechanisms. As desertification intensifies, bacterial networks exhibit a trend of decreasing connectivity density, while the proportion of positive interactions shows an overall increase across the gradient (HLA: 52.74%; ZRHA: 62.17%; WLT: 61.63%). This pattern generally aligns with the predictions of the Stress Gradient Hypothesis and is highly consistent with numerous recent findings in arid grassland ecosystems (Hong et al., 2021; Wang et al., 2022). Previous study along an aridity gradient in alpine grasslands of the Tibetan Plateau, similarly found that the complexity of bacterial, fungal, and protist co-occurrence networks significantly decreased with increasing aridity (Zhang et al., 2024a). Likewise, semi-arid grasslands of northern China demonstrated that aridity directly reduced the complexity and stability of microbial networks (Wang C. et al., 2023). The increase in the proportion of positive interactions observed in this study aligns with the phenomenon reported in these studies–that aridity leads to network simplification but may enhance cooperation among remaining taxa–indicating that enhancing cooperative relationships is a common ecological adaptation strategy for microorganisms to maintain functional stability in resource-limited desertified environments (Wang et al., 2022).

Notably, at the moderately desertified WLT site, the acidic environment (pH 6.69) was correlated with a simplified bacterial network structure while simultaneously displaying the highest proportion of positive interactions (61.63%). These findings demonstrate that pH variation can significantly influence the abundance of specific functional microbial taxa and their interaction patterns (Rousk et al., 2010; Zhong et al., 2022). The acidic environment at the WLT site in this study may have indirectly influenced resource heterogeneity by altering the soil chemical microenvironment, thereby regulating microbial interaction patterns. Further research emphasizes that soil heterogeneity plays a key role in maintaining microbial network stability: higher resource heterogeneity can promote microbial interaction complexity by providing more niches (Wang C. et al., 2023). This observation is further supported by studies indicating that under nutrient-poor conditions, microbial communities tend to strengthen mutualistic interactions to facilitate resource acquisition (Cao et al., 2022; Li Z. et al., 2024), providing theoretical foundation for the transformation of microbial interaction patterns observed in desertified environments in this study. Recent findings have further highlighted a significant positive correlation between microbial network complexity and soil multifunctionality, while decreased network robustness and increased vulnerability threaten the stability of ecosystem functions (Wang C. et al., 2023; Xiao et al., 2025).

Soil physicochemical properties significantly shape microbial network structures. Our study found that AP content was positively correlated with the proportion of positive bacterial interactions (62.17% positive connections at ZRHA site with 34.54 mg kg^–1^ AP), while fungal networks showed greater sensitivity to soil organic carbon (SOC) content (12.4–15.2 g kg^–1^). As a core environmental factor, pH exhibited universal regulatory effects (*p* < 0.01), consistent with global-scale findings that soil pH serves as a key driver of microbial community assembly (Duan et al., 2025; Zhong et al., 2022). Previous research has demonstrated that root exudates and microbial activities jointly regulate soil organic matter turnover, which may explain the underlying mechanism of fungal network responses to SOC changes (Huang et al., 2021; Whalen et al., 2021). Studies have further confirmed the importance of plant-microbe interactions for soil improvement, showing that alfalfa cultivation significantly enhances soil microbial biomass and enzyme activities, with intercropping systems demonstrating optimal effects (Mbuthia et al., 2015; Tahir et al., 2023).

The stability of microbial networks depends on the presence of keystone species, which supports the “core microbiome” theory (Liu et al., 2022). Recent studies have shown that mycorrhizal fungi serve as critical mediators in plant-microbe interaction networks by facilitating resource exchange and promoting coexistence among different plant species (Khashi u Rahman et al., 2019; Tedersoo et al., 2020; Zhang et al., 2024b). The research findings demonstrate that fungal networks in moderately-desertified WLT plots maintain an average connectivity degree of 17.11. This distinct network connectivity pattern reveals that fungal mycelial networks play an essential role in preserving the structural and functional integrity of microbial communities (Jin et al., 2024; Liang et al., 2020). Furthermore, existing evidence confirms the important role of AMF in rehabilitating desertified soils, providing additional support for the functional value of keystone taxa in stressed ecosystems (Fu et al., 2025; Li M. et al., 2024).

Collectively, our findings demonstrate that desertification gradients drive the transformation of microbial interaction networks from complex competition-cooperation equilibria toward simplified, cooperation-dominant structures through alterations in soil physicochemical properties (particularly pH and nutrient status). These insights provide important implications for ecological restoration of desertified lands: (1) microbial interaction patterns could be optimized by regulating soil pH and nutrient availability; (2) conservation and introduction of keystone species (e.g., mycorrhizal fungi with ecosystem engineering functions) should be prioritized. Future research incorporating metagenomic approaches could further elucidate the variation patterns of microbial functional genes along desertification gradients, thereby offering more precise theoretical guidance for ecological restoration practices.

### 4.3 Metabolic complementarity reveals cross-kingdom ecological synergy

This study integrates high-throughput functional prediction with soil physicochemical data to unravel how desertification gradients drive the functional differentiation of rhizosphere microbial communities in alfalfa. Despite the inherent limitations of prediction tools (e.g., PICRUSt2), which include potential biases from incomplete databases, our analysis reveals a core suite of conserved metabolic functions alongside key stress-responsive adaptations that are critically shaped by the soil environment.

A high degree of functional conservation was observed in core bacterial housekeeping processes across all sites. Pathways such as “Metabolic pathways,” “Biosynthesis of secondary metabolites,” and “Microbial metabolism in diverse environments” exhibited minimal variation, underscoring a stable foundational metabolome. Similarly, fungal communities displayed stable investments in essential functions like energy metabolism (Adenosinetriphosphatase) and genetic information processing (DNA polymerases). This suggests a resilient core microbiome capable of maintaining essential ecosystem processes like nutrient cycling across a range of desertification stresses (Jiao et al., 2019; Sun et al., 2023).

However, significant variations were observed in specific pathways directly related to environmental stress and nutrient acquisition, which were strongly correlated with gradients in soil properties. The most compelling evidence for desertification-driven adaptation was the significant enrichment of stress-response and detoxification enzymes at specific sites, with functional adaptations manifesting not only in detoxification capabilities but also in strategies for utilizing complex carbon sources. The moderately desertified WLT site (pH = 6.69) exhibited unique adaptive characteristics: on one hand, detoxifying enzymes such as unspecific monooxygenase (1.94%), choline dehydrogenase (1.13%), and salicylate 1-monooxygenase (0.97%) were enriched, suggesting that the microbial community has evolved powerful adaptive capabilities (Coleine et al., 2024); on the other hand, the levels of carbohydrate-degrading enzymes such as L-arabinose isomerase (2.92%) and glucan 1,4-alpha-glucosidase (2.74%) were also markedly elevated at this site, reflecting a microbial investment in versatile enzyme systems to access limited and complex carbon sources under moderate desertification conditions (Zhu et al., 2022). This stands in stark contrast to the nutrient-rich non-desertified sites (ZRHC, ZLT)–although these sites possessed higher SOC (11.41–11.62 g ⋅ kg^–1^) and C/N ratios (11.7–15.7), they did not exhibit the same degree of specific enzyme enrichment, suggesting that organic matter decomposition there is likely undertaken by other microbial consortium.

In conclusion, desertification does not eradicate core microbial metabolic functions but rather drives a strategic functional divergence. It selects for a stress-responsive “specialist” phenotype in harsh environments (e.g., WLT with low pH). In contrast, stable, resource-rich environments maintain a core “generalist” community. These findings provide a mechanistic understanding of microbial adaptation to desertification.

### 4.4 Harnessing rhizosphere microbiome for sustainable soil management in arid agroecosystems

This study provides important theoretical foundations and practical guidance for soil health management in the agro-pastoral ecotone of arid and semi-arid regions from a microbiome perspective. Our findings demonstrate significant correlations between the structural and functional characteristics of alfalfa rhizosphere microbial communities and soil environmental factors, offering a scientific basis for developing microbiome-based precision agriculture technologies. Specifically, we propose two key strategies: First, the utilization of biocontrol microorganisms (e.g., *Bacillus subtilis*) to suppress the over-proliferation of pathogenic fungi (e.g., *Alternaria* spp.). Second, the construction of modular synthetic microbial communities (SynComs) based on identified keystone functional microorganisms (including *Sphingomonas* spp., *Bacillus subtilis*, and efficient nitrogen-fixing rhizobia) (Bai et al., 2015; Li Y. et al., 2023). Through optimized strain combinations and inoculation methods, these SynComs can simultaneously establish efficient nitrogen fixation systems and maintain rhizosphere microecological balance, enabling precise regulation. These findings establish an important foundation for developing next-generation microbiome technologies.

## 5 Conclusion

This study elucidates the structural and functional adaptation mechanisms of alfalfa rhizosphere microbial communities along a desertification gradient. Microbial community composition analysis revealed Proteobacteria (27.22%–37.64%), Actinobacteria (16.38%–28.56%), and Acidobacteria (10.89%–27.00%) as the dominant bacterial phyla, while Ascomycota (69.7%–85.5%) predominated among fungi. Soil analysis demonstrated that physicochemical parameters explained 33% of bacterial communities and 28% fungal communities variation, with pH and available phosphorus (34.54 mg kg^–1^) emerging as key regulators of microbial interactions. Functional prediction analyses revealed that desertification gradients drove microbial adaptive functional differentiation from conserved core metabolism (e.g., amino acid biosynthesis, <1% variation) to site-specific stress-response pathways (e.g., monooxygenase enrichment up to 1.94% in WLT), with metabolic divergence primarily regulated by soil pH and nutrient availability. These findings provide critical insights for desertified land restoration, recommending a gradient rehabilitation strategy that includes drought-tolerant strain inoculation, optimized organic matter amendment, and balanced soil pH and nutrient management.

## Data Availability

The datasets presented in this study can be found in the NCBI Sequence Read Archive (SRA) under BioProject accession numbers PRJNA1303413, http://www.ncbi.nlm.nih.gov/bioproject/1303413 (for prokaryotes) and PRJNA1303431, http://www.ncbi.nlm.nih.gov/bioproject/1303431 (for fungi).

## Footnotes

1
https://huttenhower.sph.harvard.edu/galaxy

2
https://inap.denglab.org.cn/
