# Supplementary material for: Desertification gradients shape Medicago sativa rhizosphere microbiomes in inner Mongolia’s agro-pastoral ecotone
Source: Front Microbiol. 2025 Sep 22;16:1651717. doi: 10.3389/fmicb.2025.1651717 (PMC12500245; doi:10.3389/fmicb.2025.1651717)
Supplement: Supplementary file 1 [file Supplementary_file_1.docx]

Supplementary Material

# Supplementary Figures and Tables

## Supplementary Figures


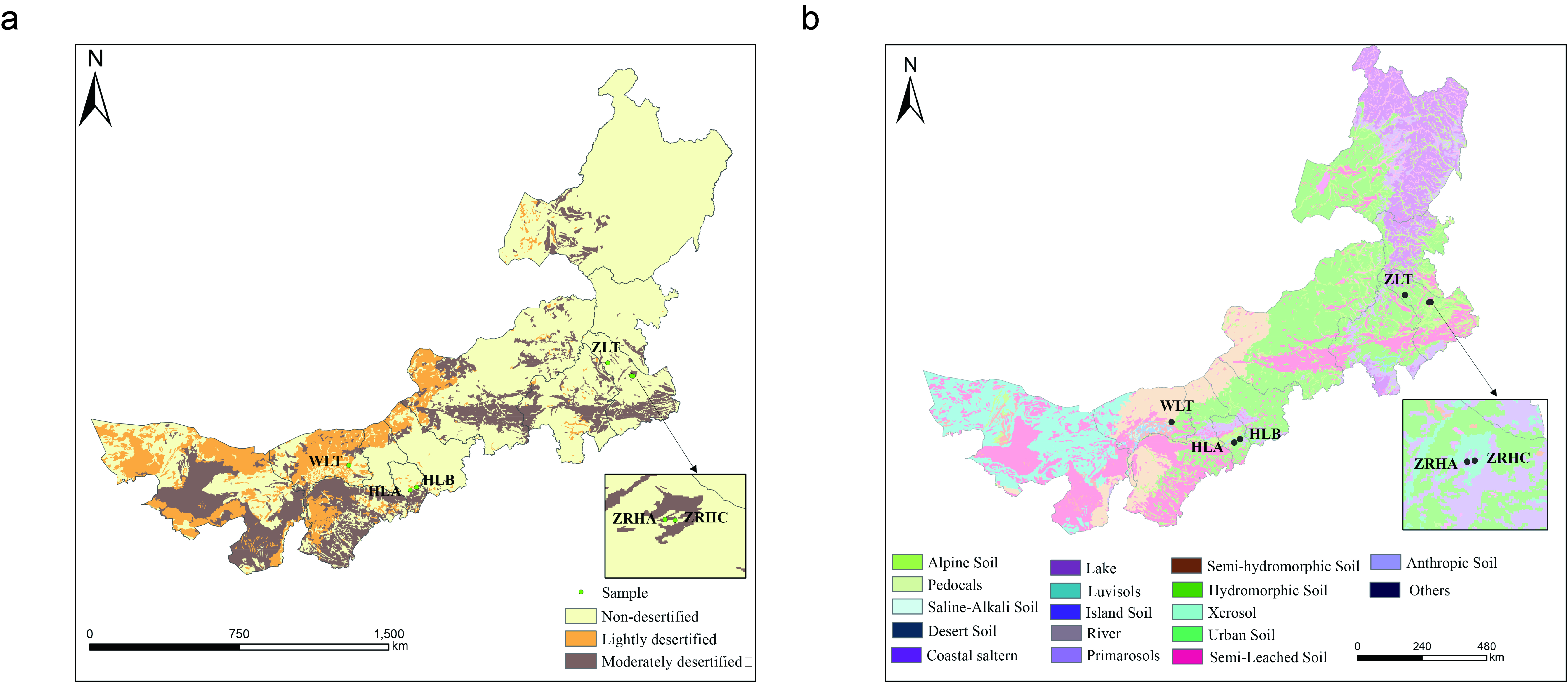


**Supplementary Figure 1.** Distribution map across six sampling sites in Inner Mongolia . (**a**)desertification level.(**b**)Soil types.
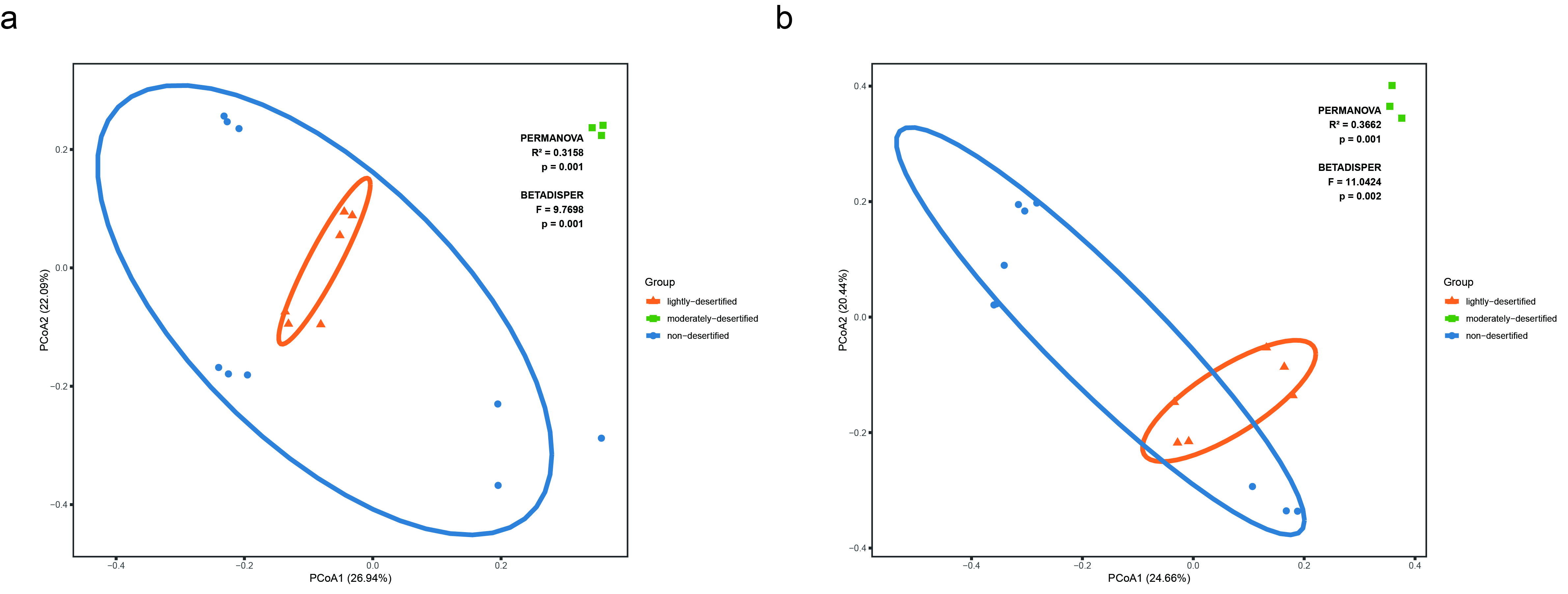


**Supplementary Figure 2.Principal coordinate analysis derived from NDVI (Normalized Difference Vegetation Index.NDVI)values (a)bacterial community based desertification gradient (Bray-Curtis distance).(b)fungal community based desertification gradient (Bray-Curtis distance).The statistical significance of beta diversity differences between groups was assessed using PERMANOVA and BETADISPER**


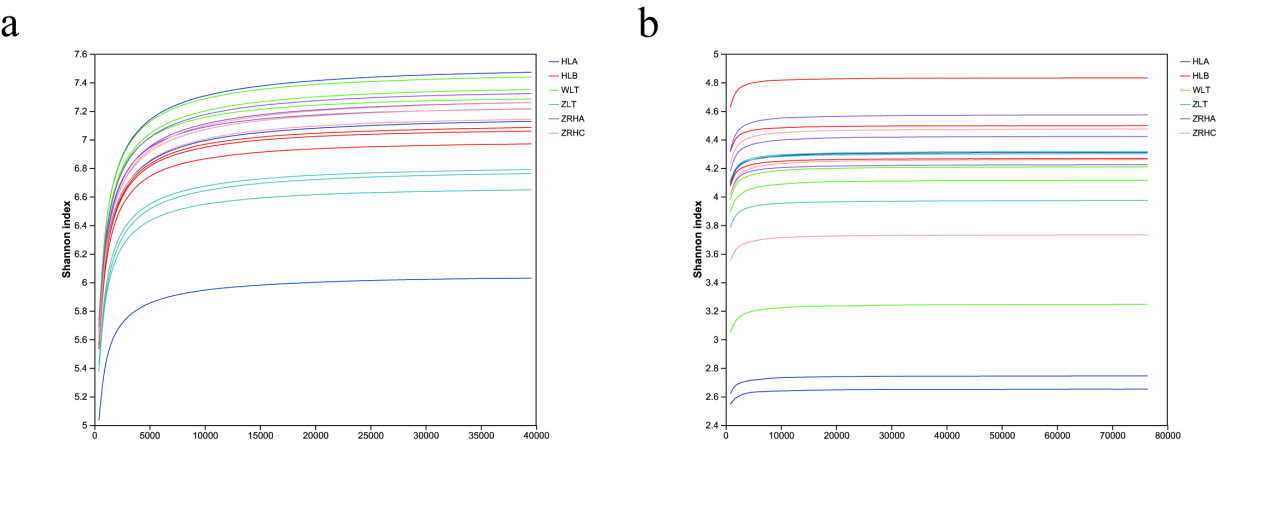


**Supplementary Figure 3.** Distribution map across six sampling sites in Inner Mongolia . (a)desertification level.(b)Soil types.


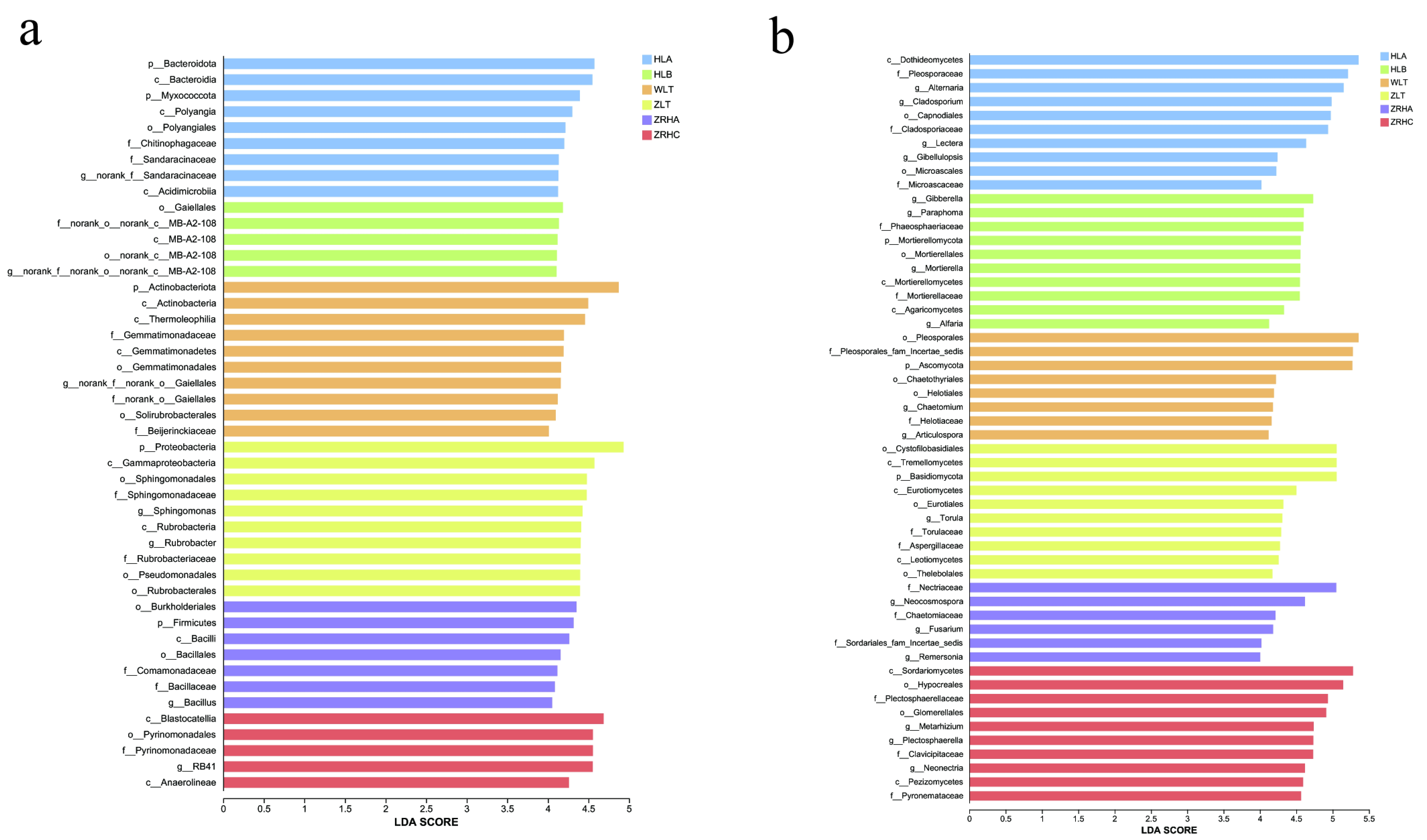


**Supplementary Figure 4.** Cladogramis used to show taxonomic distribution of marker species in each group of samples. (a). Bacteria; (b). Fungi. Multiple testing correction was performed using the Benjamini-Hochberg FDR, *q*<0.05，LDA scores ＞4.0.


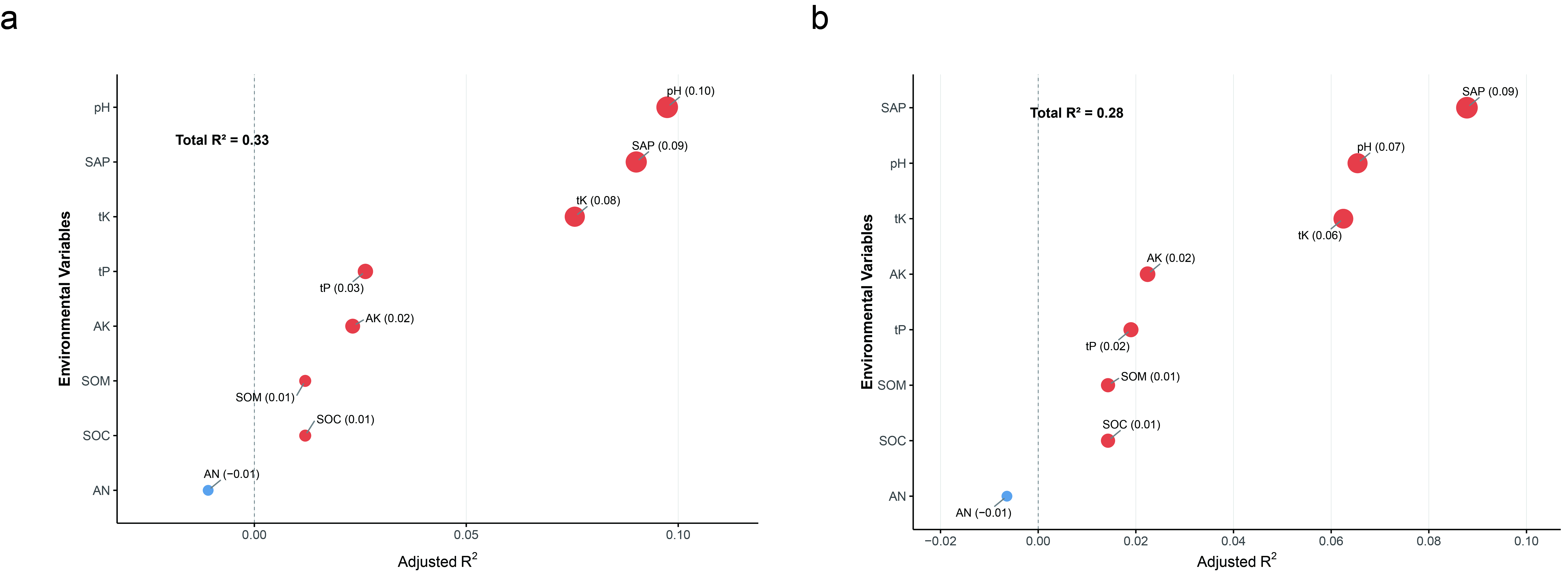


**Supplementary Figure 5.** Variance partitioning analysis (VPA) of rhizosphere microbial communities.(a). Bacteria; (b). Fungi. Significance tested by RDA permutation test (*p* < 0.05, 999 permutations).

## Supplementary Tables

**Supplementary Table 1.** Basic spatial geographic parameters information of the study site.

| Sample site | Site code | Longitude | Latitude | Climate type | Sampling time |  |
| --- | --- | --- | --- | --- | --- | --- |
|  |  |  |  |  |  |  |
| Tongliao city | ZRHA | 121°35′58.35″ | 44°12′5.293″ | semi-arid | 2023.9.12 |  |
| Tongliao city | ZRHC | 121°31′47.4″ | 44°12′22.321″ | semi-arid | 2023.9.12 |  |
| Tongliao city | ZLT | 120°27′23.417″ | 44°37′16.555″ | semi-arid | 2023.9.13 |  |
| Hohhot city | HLA | 111°33′3.801 | 40°23′3.754 | semi-arid | 2023.9.14 |  |
| Hohhot city | HLB | 111°50′6.714″ | 40°29′3.5916″ | semi-arid | 2023.9.14 |  |
| Bayannaoer city | WLT | 108°46′48.971″ | 41°13′59.241″ | arid | 2023.9.16 |  |

**Supplementary Table 2.** The network indices of bacterial communities.

| taxa | Number of node | Number of edge | Average  degree | Modularity | Positivity edge(%) | Negitivity edge(%) |
| --- | --- | --- | --- | --- | --- | --- |
| HLA | 62 | 1003 | 32.355 | 0.147 | 52.74 | 47.26 |
| HLB | 79 | 769 | 19.443 | 0.272 | 53.91 | 46.09 |
| WLT | 89 | 607 | 13.64 | 0.26 | 61.63 | 38.39 |
| ZLT | 57 | 636 | 22.316 | 0.215 | 71.7 | 28.3 |
| ZRHA | 84 | 1255 | 29.881 | 0.238 | 62.17 | 32.83 |
| ZRHC | 77 | 881 | 22.883 | 0.294 | 69.92 | 30.08 |

**Supplementary Table 3.** The network indices of fungal communities.

| taxa | Number of node | Number of edge | Average  degree | Modularity | Positivity edge(%) | Negitivity edge(%) |
| --- | --- | --- | --- | --- | --- | --- |
| HLA | 45 | 563 | 23.957 | 0.23 | 50.09 | 49.91 |
| HLB | 35 | 207 | 11.829 | 0.251 | 50.72 | 49.28 |
| WLT | 47 | 402 | 17.106 | 0.218 | 54.23 | 45.77 |
| ZLT | 55 | 672 | 24.436 | 0 .209 | 50.6 | 49.4 |
| ZRHA | 61 | 747 | 24.492 | 0.251 | 59.57 | 40.43 |
| ZRHC | 57 | 815 | 28.596 | 0.163 | 53.5 | 46.5 |

**Supplementary Table 4.** Soil physical of soil samples from the *Medicago sativa.*

|  | HLA | HLB | WLT | ZLT | ZRHA | ZRHC |
| --- | --- | --- | --- | --- | --- | --- |
| pH | 8.57±0.1a | 8.56±0.07a | 6.69±0.16b | 8.7033±0.02a | 8.5±0.25a | 8.67±0.02a |
| tP (g•kg^-1)^ | 1.0217±0.1a | 1.5073±0.27a | 1.5907±0.46a | 1.0223±0.14a | 0.197±0.01b | 0.2447±0.002b |
| tK(g•kg^-1)^ | 8.15±0.16d | 11.0967±0.50cd | 14.7667±0.43b | 18.9933±2.53a | 13.5017±0.09bc | 15.514±0.07b |
| AN(mg•kg^-1)^ | 43.4±3.05 | 50.4±3.37 | 43.75±7.48 | 47.7167±8.53 | 53.6667±1.22 | 54.3667±0.42 |
| SAP(mg•kg^-1)^ | 25.6337±3.08bc | 21.3913±1.86c | 25.4627±0.53bc | 3.0857±0.37d | 34.5443±0.74a | 28.879±0.69b |
| AK(mg•kg^-1)^ | 45.6667±9.8c | 60.0333±7.67bc | 86.9667±16.58ab | 92.6333±13.46ab | 108.3±1.38a | 106.8667±3.00a |
| SOC(g•kg^-1)^ | 6.492±0.90b | 6.1993±1.37b | 9.3757±0.53b | 11.4107±2.21a | 11.0733±1.09a | 11.62±0.75a |
| SOM(g•kg^-1)^ | 11.1923±1.55b | 10.688±2.36b | 16.1637±0.91ab | 19.671±3.81a | 19.0967±1.87a | 20.0333±1.30a |
| N% | 0.0583±0.007c | 0.085±0.006b | 0.0963±0.002b | 0.1247±0.01a | 0.1077±0.01ab | 0.106±0.01ab |
| C% | 0.5523±0.04c | 0.7187±0.06c | 0.627±0.03c | 1.432±0.04b | 1.6997±0.11a | 1.6673±0.01a |
| C/N | 9.6809±0.59bc | 8.4852±0.49cd | 6.4961±0.27d | 11.7188±1.30b | 15.8859±0.63a | 15.7481±0.33a |

**Supplementary Table 5.**Variance inflation factors (VIF) of predictor variables(VIF < 5: Collinearity is not severe;5 ≤ VIF < 10: Moderate multicollinearity exists;VIF ≥ 10: Indicates severe multicollinearity).

| Variable | VIF |
| --- | --- |
| pH | 3.73231138813912 |
| tP | 3.87335075779444 |
| tK | 3.05792196009266 |
| AN | 1.96175116902051 |
| SAP | 3.12750065569384 |
| AK | 2.67121782156233 |
| SOC | 2.32952463089218 |
| SOM | 2.32978999297715 |
